# Synthesis and Antibacterial Evaluation of an Indole Triazole Conjugate with In Silico Evidence of Allosteric Binding to Penicillin-Binding Protein 2a

**DOI:** 10.3390/pharmaceutics17081013

**Published:** 2025-08-03

**Authors:** Vidyasrilekha Sanapalli, Bharat Kumar Reddy Sanapalli, Afzal Azam Mohammed

**Affiliations:** 1 Department of Pharmaceutical Chemistry, JSS College of Pharmacy, JSS Academy of Higher Education & Research, Ooty, The Nilgiris 643001, TN, India; afzal@jssuni.edu.in; 2 Department of Pharmaceutical Chemistry, School of Pharmacy & Technology Management, SVKM’s Narsee Monjee Institute of Management Studies (NMIMS) Deemed-to-be-University, Jadcherla, Hyderabad 509301, TS, India; 3 Department of Pharmaceutics, JSS College of Pharmacy, JSS Academy of Higher Education & Research, Ooty, The Nilgiris 643001 TN, India; bharathsanapalli@yahoo.in; 4 Department of Pharmacology, School of Pharmacy & Technology Management, SVKM’s Narsee Monjee Institute of Management Studies (NMIMS) Deemed-to-be-University, Jadcherla, Hyderabad 509301, TS, India; 5 Department of Pharmaceutical Chemistry, JSS College of Pharmacy, JSS Academy of Technical Education, Noida 201301, UP, India

**Keywords:** antibacterial resistance, indole triazole conjugate, antibacterial activity, molecular docking, molecular dynamics simulation studies

## Abstract

**Background**: Antibacterial resistance (ABR) poses a major challenge to global health, with methicillin-resistant *Staphylococcus aureus* (MRSA) being one of the prominent multidrug-resistant strains. MRSA has developed resistance through the expression of Penicillin-Binding Protein 2a (PBP2a), a key transpeptidase enzyme involved in bacterial cell wall biosynthesis. **Objectives**: The objective was to design and characterize a novel small-molecule inhibitor targeting PBP2a as a strategy to combat MRSA. **Methods**: We synthesized a new indole triazole conjugate (ITC) using eco-friendly and click chemistry approaches. In vitro antibacterial tests were performed against a panel of strains to evaluate the ITC antibacterial potential. Further, a series of in silico evaluations like molecular docking, MD simulations, free energy landscape (FEL), and principal component analysis (PCA) using the crystal structure of PBP2a (PDB ID: 4CJN), in order to predict the mechanism of action, binding mode, structural stability, and energetic profile of the 4CJN-ITC complex. **Results**: The compound ITC exhibited noteworthy antibacterial activity, which effectively inhibited the selected strains. Binding score and energy calculations demonstrated high affinity of ITC for the allosteric site of PBP2a and significant interactions responsible for complex stability during MD simulations. Further, FEL and PCA provided insights into the conformational behavior of ITC. These results gave the structural clues for the inhibitory action of ITC on the PBP2a. **Conclusions**: The integrated in vitro and in silico studies corroborate the potential of ITC as a promising developmental lead targeting PBP2a in MRSA. This study demonstrates the potential usage of rational drug design approaches in addressing therapeutic needs related to ABR.

## 1. Introduction

Antibacterial resistance (ABR) is an escalating threat to public health globally, driven by the rapid dissemination of resistance mechanisms among nosocomial pathogens. This phenomenon jeopardizes the effectiveness of conventional antibiotic treatments for common infectious diseases [1,2]. Despite continuous efforts to develop new antibiotics, the clinical utility of novel agents is often compromised by the rapid evolution of bacterial resistance. Historically, the discovery of antibacterial agents in the twentieth century largely relied on modifying the synthetic structures of existing antibiotics [3]. However, contemporary strategies now focus on the development of structurally distinct antibiotic classes that target clinically validated pathways to mitigate resistance. Although a broad range of antibiotics are available in the market, the majority act on a limited set of bacterial cellular functions: cell wall formation [4], folate metabolism [5], protein synthesis [6], and nucleic acid replication [7]. The evolutionary adaptability of bacterial receptor proteins under antibacterial pressure facilitates resistance, predominantly through horizontal gene transfer and genetic mutations [3]. One such key target is Penicillin-Binding Protein 2a (PBP2a), a membrane-bound bacterial transpeptidase enzyme and a critical biological macromolecule involved in β-lactam resistance, particularly in methicillin-resistant *Staphylococcus aureus* (MRSA). Repeated exposure of bacterial pathogens to antibiotics promotes genetic mutations, thereby intensifying the ABR crisis [8,9]. This underlines the necessity for novel antibiotics with diverse pharmacophoric features to circumvent cross-resistance and enhance therapeutic outcomes.

Indoles and triazoles, aromatic heterocyclic structures, have attracted significant attention for drug discovery owing to their diverse biological activity and enormous pharmaceutical potential. Indoles and triazole-based derivatives have significant activity against various microbes, such as fungi and bacteria [10,11,12]. Indole, a privileged scaffold, exhibits a broad structural and mechanistic diversity, enabling it to interact with multiple bacterial enzymes with high affinity. Because of the diverse biological functions of the Indole, including stress response, nutrient acquisition, cellular growth, motility, biofilm formation, and virulence, it has been established as a promising scaffold for antibacterial drug discovery [13,14]. As a consequence, indole derivatives are endowed with a diversity of pharmacological activities, including antibacterial, antifungal, antiviral, antimalarial, antitubercular, and anticancer activities, and can thus be considered highly potent molecules [15,16,17,18]. In addition, indole and its derivatives exhibited remarkable activity against a range of clinically important strains of bacteria [19]. These compounds are promising leads owing to their structural versatility and capacity to engage multiple pathways in bacteria. They have a therapeutic potential against resistant strains because of their ability to intervene with vital processes in the bacteria [13,14,17,20,21]. Similarly, the triazoles, which represent a privileged heterocyclic pharmacophore, are capable of a variety of noncovalent interactions with many biological targets [22,23,24,25,26,27]. These properties account for their broad-spectrum antimicrobial activity [28,29,30,31,32]. Therefore, the combination of indole and triazole moieties has become a valuable approach toward improved antibacterial activity through the simultaneous suppression of multiple bacterial targets [33,34]. A combination of indole and triazole exhibited synergistic effects that may enhance drug efficacy and minimize resistance development [34,35,36,37,38].

In the present work, we have developed an indole triazole conjugate (**ITC**) via green and click chemistry strategies. Green chemistry principles were followed (selected reagents being less hazardous materials, energy-saving protocols), and regioselective syntheses and mild reaction conditions were achieved using click chemistry.

The inhibitory efficacy of ITC against a selected panel of bacterial strains was evaluated using in vitro assays. Preliminary results demonstrated promising inhibitory effects, indicating its potential for the advancement of newer antibacterial agents. Mechanistic insights into the mode of bacterial inhibition were further investigated using computational methodologies. In early-stage antibacterial drug discovery, compound promiscuity and non-specific binding are important concerns that must be critically evaluated to avoid misleading efficacy outcomes. Promiscuous compounds often exhibit broad-spectrum activity through non-selective interactions, which may result in off-target effects, toxicity, and ultimately poor clinical translation. This is particularly relevant for in silico studies, where docking to multiple potential targets can artificially inflate predicted efficacy. Therefore, careful consideration of binding specificity and drug-likeness is essential. In our study, ITC was evaluated with respect to its binding behavior at the well-characterized allosteric site of PBP2a using validated docking protocols, and its chemical purity and analytical integrity were confirmed prior to biological testing. To ameliorate the affinity and interaction profile of the ITC, in silico studies were conducted. In addition, MM-PBSA-based calculation of free energy was computed to provide a deeper understanding of complex stability. A molecular dynamics (MD) simulation study was used to analyze the dynamics, stabilities, and conformational behavior over a longer time scale, which provided greater insight into the molecular interactions under physiological conditions. The combined experimental and computational findings revealed key interactions responsible for antibacterial efficacy, offering a comprehensive insight into the ITC’s mechanism of action. This study demonstrates the promise of ITC as an antibacterial agent and testifies to the combined contribution of green synthesis and computational chemistry for the rational delivery of bioactive molecules.

## 2. Materials and Methods

### 2.1. Chemistry

#### 2.1.1. Preparation of 1-Azido-4-methoxybenzene (AZMB)

(4-Methoxyphenyl)methanamine (4-MPM) (1 g, 7.29 mmol) was solubilized in 25 mL of 4 M HCl and subjected to 0 °C cooling. Sodium nitrate (0.603 g, 8.7 mmol) was added dropwise over 10 min, followed by stirring for 30 min. Further, sodium azide (0.702 g, 10.8 mmol) was added in portions to the reaction mixture and stirred for 30 min at 25 °C. Later, the mixture was extracted with ethyl acetate (C_2_H_5_OH), dried, and the crude product was cooled and stored.

#### 2.1.2. Preparation of 3-(((2-(4-Methoxyphenyl)-2H-1,2,3-triazol-4-yl) methoxy)methyl)-1-tosyl-1H-indole (ITC)

(i) Preparation of 1-tosyl-1H-indole-3-carbaldehyde (**C**)

Indole-3-carbaldehyde (**A**) (1.5 g, 10 mmol), triethylamine (TEA) (2 g, 20 mmol), and 4-dimethylaminopyridine (DMAP) (0.122 g, 1 mmol) were stirred together in anhydrous DCM (20 mL) at 25 °C. A solution of p-toluenesulfonyl chloride (**B**) (2.86 g, 15 mmol) in anhydrous DCM (20 mL) was added dropwise to the above mixture and stirred for 3.5 h at room temperature, monitored by TLC. Furthermore, the mixture was cooled and extracted with C_2_H_5_OH, washed with brine and 1 M HCl, concentrated in vacuo, followed by purification using column chromatography (CC). Yield: 70%

(ii) Preparation of (1-tosyl-1H-indol-3-yl) methanol (**D**)

A solution of **C** (0.5 g, 1.67 mmol) in CH_3_OH (10 mL) was added with NaBH_4_ (0.064 mg, 1.67 mmol) in portions and stirred at 0 °C for 0.5 h. The mixture was extracted with C_2_H_5_OH, washed with brine, and dried over Na_2_SO_4_ to obtain the crude product. Yield: 98%

(iii) Preparation of 3-(chloromethyl)-1-tosyl-1H-indole (**E**)

At 0 °C under constant stirring, intermediate **D** (0.502 g, 1.66 mmol) in anhydrous DCM (10 mL), TEA (0.336 g, 3.33 mmol), and methane sulfonyl chloride (0.229 g, 1.99 mmol) were added, followed by stirring for 30 min. The mixture was extracted with DCM, washed with brine, and dried over Na_2_SO_4_ to obtain the crude product. Yield: 75%

(iv) Preparation 3-((prop-2-yn-1-yloxy) methyl)-1-tosyl-1H-indole (**F**)

K_2_CO_3_ (1.58 g, 11.5 mmol) and propargyl alcohol (0.262 g, 4.6 mmol) were added to a solution **E** (1.5 g, 4.6 mmol) in CH_3_CN (20 mL) and stirred for 12 h at 80 °C, followed by extraction with C_2_H_5_OH, followed by washing with brine, concentrated under vacuum and purified by CC. Yield: 85%

(v) Preparation of **ITC**

**Figure 1:**
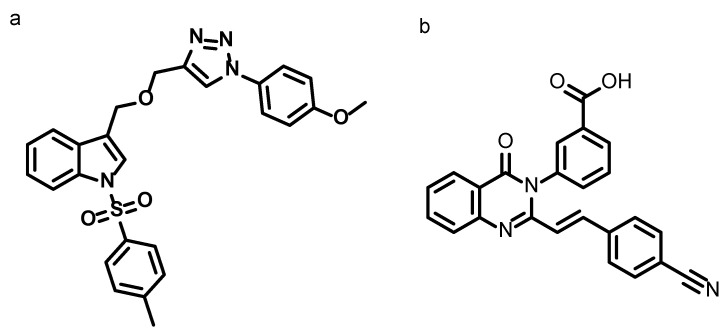
Chemical structures of (**a**) synthesized compound 3-(((2-(4-methoxyphenyl)-2H-1,2,3-triazol-4-yl) methoxy) methyl)-1-tosyl-1H-indole and (**b**) co-crystallized ligand (E)-3-(2-(4-cyanostyryl)-4-oxoquinazolin-3(4H)-yl)benzoic acid of PBP2a (PDB ID: 4CJN).

A mixture of **F** (0.1 g, 0.29 mmol) in water: t-butanol (10:1, */*) was stirred in an RBF. TEA (0.676 g, 6.7 mmol) was added in portions and stirred for 2 h at room temperature. Then, AZMB (0.546 g, 3.35 mmol) and CuI (0.0315 g, 0.167 mmol) were introduced into the above mixture and stirred for 24 h at room temperature. The mixture was extracted twice with C_2_H_5_OH and dried over Na_2_SO_4_. The obtained product (Figure 1a) was then purified by CC.

Molecular Formula: C_26_H_25_N_4_O_4_S.
Molecular Weight (*m*/*z*): 488.1518.
Observed Mass (*m*/*z*): 489.1596 [M + 1].
Melting Point (°C): 189.
Yield: 67.98%.

FT-IR (cm^−1^): 3005, 2987 (aromatic stretching C-H vibration peaks), 3106 (C-H stretching, triazole ring), 3025 and 3045 (indole symmetric and asymmetric C-H stretching vibration peaks), 2967 (CH_3_ stretching vibration), 2845 (CH_2_ stretching vibration), 1239 (ether O-C stretching), 1165 and 1136 (SO_2_ symmetric stretching vibrations), 1458 (CH_3_ bending vibration).

^1^H NMR (500 MHz, DMSO-d_6_) δ 8.71 (s, 1H, C-H triazole), 7.93 (d, *J* = 8.3 Hz, 1H), 7.87 (s, 1H), 7.85 (d, *J* = 4.9 Hz, 2H), 7.81 (t, 1H), 7.79 (d, *J* = 2.1 Hz, 1H), 7.62 (d, *J* = 7.8 Hz, 1H), 7.37 (d, *J* = 7.9 Hz, 3H), 7.35 (d, *J* = 0.9 Hz, 1H), 7.27 (t, *J* = 7.5 Hz, 1H), 7.15 (d, *J* = 2.1 Hz, 1H), 7.13 (d, *J* = 2.1 Hz, 1H), 4.72 (s, 2H, CH_2_), 4.64 (s, 2H, CH_2_), 3.84 (s, 3H, OCH_3_), 2.30 (s, 3H, CH_3_).^13^C NMR (126 MHz, DMSO-d_6_) δ 159.75 (C-O-C, aromatic ring), 159.72 (C-N, triazole), 146.01, 145.97, 145.14, 135.08, 134.70, 134.62, 130.72, 130.58, 130.19, 127.16, 125.84, 125.46, 123.88, 122.74, 122.31, 120.88, 120.03, 115.39, 115.35, 113.69, 63.42 (CH_2_), 63.10 (CH_2_), 56.10 (OCH_3_), 22.02 (CH_3_).

### 2.2. In Vitro Studies

#### 2.2.1. Minimum Inhibitory Concentration (MIC_50_)

Selective bacterial strains were screened against ITC for their antibacterial activity. MIC_50_ of ITC was performed in Hi-media using a micro-broth dilution method as per CLSI guidelines. The Gram negative bacteria (GNB) used were (*Escherichia coli* (EC) (NCIM 2567), *Klebsiella pneumonia* (KP) (NCIM 2706), *Pseudomonas aeruginosa* (PA) (NCIM2036), *Salmonella typhi* (ST) (NCIM 2501)), Gram positive bacteria (GPB) (*Staphylococcus aureus* (SA) (NCIM5022), *Staphylococcus epidermis* (SE) (NCIM 2493), *Bacillus subtilis* (BS) (NCIM2545), Methicillin resistant *Staphylococcus aureus* (MRSA-(ATCC 43300 and NCIM 5021)) and the concentration range used for antibacterial screening is 0.015 to 20 μg/mL. The bacterial culture was diluted to 1:1000 nutrient broth to obtain 0.5 McFarland. In the study, ciprofloxacin (CIP) and DMSO were used as positive and negative controls, respectively. Each of the 96-well plates was filled with **ITC** (50 μL), followed by 100 μL of bacterial culture, and then serially diluted. Following inoculation, the wells were incubated (37 °C; 20–24 h), except for PA (37 °C; 24–28 h). Measured absorbance (600 nm) was used to calculate MIC_50_ values (μg/mL) [39]. MIC_50_ was interpolated from dose–response curves using 4-parameter logistic regression as the concentration achieving 50% inhibition.

#### 2.2.2. Minimum Bactericidal Concentration (MBC)

The bactericidal nature of ITC was assessed using MBC. The procedure, which was already reported, was used for MBC of the compound against selected pathogenic bacteria. Nutrient agar plates were inoculated with ITC (concentrations above MIC_50_). The plates were incubated for 24 h at 37 °C, and colony counting was performed with the original level of inoculum. MBC is the concentration that caused the bacterial count to be reduced by ≥99% from its original number [40,41,42].

#### 2.2.3. Time–Kill Kinetics

The study was performed using the standard protocol, in which 0.5× MIC_50_ and 1× MIC_50_ concentrations of the ITC were used. After preparing the sample concentrations with bacterial culture, incubation was conducted at 37 °C. From the culture, 50 μL of aliquots for every 0, 3, 6, 12, 24 h were inoculated and incubated for 24 h at 37 °C. CIP was used as a positive control, whereas DMSO was a negative control. Nutrient agar and nutrient broth were also incubated to avoid bias in the results. The CFU of the test organism seen on the plate was measured. A graph displaying the log CFU/mL values plotted against time was generated [43,44].

#### 2.2.4. Cell Cytotoxicity

Cytotoxicity of ITC was determined using an MTT assay employing Vero cells. Cells (10^3^/well) were plated in a 96-well plate for 24 h, and ITC (100–5 mg/L) was added with 5% CO_2_ atmosphere and incubated for 72 h. MTT (5 mg/L) was introduced and incubated for 4 h, followed by formazan solubilization with DMSO. Doxorubicin served as a positive control. CC_50_ was determined at 540 nm of optical density [45].

#### 2.2.5. Post Antibiotic Effect

The overnight culture of the test organism (10^5^ CFU/mL) in MHBII was exposed to 1× and 10× MIC_50_ of ITC and incubated at 37 °C for either 1 or 2 h. The culture was centrifuged and splashed twice to eliminate residual ITC sample with pre-warmed MHBII. Cells were washed and resuspended in MHBII (without drug) and incubated, and hourly samples were serially diluted and plated for CFU counts [46]. The PAE was calculated as

(1) PAE=T−C

where T is the time needed for the drug-treated culture to increase by 1 log CFU/mL following drug removal, and C is the time for the untreated control.

#### 2.2.6. Synergy Testing

The checkerboard assay was used to assess ITC synergy with FDA-approved drugs. Stock two-fold dilutions of each antibiotic (2XMIC) were prepared in the testing medium. Antibiotic dilutions from 0.03 to 64 mg/mL in 96-well plates were plotted along the abscissa, while ITC was serially diluted along the ordinate (0.03 to 4 mg/mL). A 100 mL bacterial inoculum (~106 CFU/mL) was added and incubated aerobically for 24 h at 37 °C [47,48,49]. The ∑FICs (fractional inhibitory concentrations) were computed:

(2) ∑FIC = FIC A + FIC B

FIC A = MIC of drug A in the combination with FDA-approved drug/MIC of drug A alone;

FIC B = MIC of drug B in the combination with FDA-approved drug/MIC of drug B alone.

Synergy was defined as ∑FICs ≤ 0.5, indifference as >0.5 to 4, and antagonism as >4.

#### 2.2.7. Antibiofilm Activity

An antibiofilm activity assessment of **ITC** was performed using a 96-well polystyrene plate assay. The test organism was cultivated overnight in 1% tryptic soy broth (TSB) at 37 °C with agitation (180 RPM), followed by dilution (1:100) in fresh TSB. In total, 0.2 mL of the diluted culture was dispensed into each well and incubated statically for 48 h at 37 °C. Subsequently, the medium was removed and washed three times with PBS (pH 7.4) to eliminate planktonic bacteria. New TSB supplemented with various concentrations of ITC treatment was filled into the wells and incubated, followed by washing thrice with PBS. The formed biofilms were secured with heat (incubating 1 h, 60 °C), and stained using crystal violet solution (0.06%) for 10 min. The biofilm quantification was carried out by eluting with 30% acetic acid (0.2 mL), and the absorbance was recorded at 600 nm with the help of a microtiter plate reader. This method provides a reliable estimation of biofilm biomass based on the retention and subsequent solubilization of the stain [50].

### 2.3. In Silico Studies

#### 2.3.1. Molecular Docking

The crystal structure of Penicillin-Binding Protein 2a (PBP2a) from *Staphylococcus aureus* (PDB ID: 4CJN; resolution: 1.95 Å) was retrieved from the Protein Data Bank. PBP2a is a high-molecular-weight, membrane-associated bacterial transpeptidase enzyme that functions as a critical biological macromolecular target in β-lactam-resistant strains of MRSA. This enzyme confers resistance through active site Ser403, Lys406, and Asn464, and other residues present in the transpeptidase domain. The N-terminal (27–138 residues) and allosteric site are mainly involved in structural reconfiguration and substrate recognition. The crystal structure revealed that the bound ligand at a novel allosteric site induced conformational changes in three loops, indicative of promising allosteric regulation of the transpeptidase activity. The allosteric mechanism represents deeper insights into the design of non-β-lactam inhibitors, counteracting methicillin resistance by targeting conformational modulation rather than active pocket binding alone.

The docking of ligands against the target protein was performed using AutoDock Vina v1.1.2. Firstly, the Open Babel program was used to generate the 3D structures of the ligand (ITC) for docking after its design in ChemDraw v23.1.1.3. The 3D coordinates were generated and minimized energy using the MMFF94 force field and saved in PDBQT format. The crystal structure of the protein was prepared by removing water molecules, bound ligands, and hetero atoms. Polar atoms were added and saved in PDBQT format. The docking protocol was developed wherein the search space and grid box were set to be 60 × 60 × 60, encompassing the allosteric binding site. A co-crystallized (CCD) ligand (Figure 1b) was docked into to binding pocket of 4CJN to validate the docking protocol and compare the binding pose to that observed in the experimental crystal structure. For reproducibility and reliability, the docking was performed in triplicate, and the affinities of docking poses were evaluated [51]. Selected best binding pose was selected for further analysis. Binding modes and ligand–receptor interactions were visualized using PyMOL software [52]. The docking score reported in kcal/mol, indicates an estimate of binding propensity, not a true thermodynamic binding affinity. However, these values are employed to prioritize potential binding interactions and require further validation.

#### 2.3.2. MD Simulation Study

MD simulation was carried out on PBP2a from MRSA co-crystallized with quinazolinone ligand (PDB ID: 4CJN). The ligands ITC and CCD, coupled with the 4CJN protein (docked complexes), were selected for further evaluation. Ligand topology parameters were retrieved from the ATB server, and hydrogen atoms were added by using the pdb2gmx module of GROMACS 2018.1. The system first minimized the energy with respect to a vacuum state by steepest descent for 1500 steps. These compounds were subsequently solvated in a cubic periodic box filled with the SPCE (simple point charge) water model, and the system was neutralized afterward (0.15 M NaCl to mimic physiological ionic conditions). This methodology was adapted from an earlier described protocol [53]. A 100 ns simulation run in the NPT and NVT ensembles was conducted for each equilibrated structure and saved every 10 ps. Various parameters in terms of structural dynamics involving root-mean square deviation (RMSD), root-mean square fluctuation (RMSF), radius-of-gyration (Rg), solvent-accessible-surface area (SASA), and hydrogen bonding interactions were analyzed using GROMACS tools based on the simulation trajectory [54]. Furthermore, free energy calculation was performed for 4CJN-ITC and 4CJN-CCD complexes using the MM-PBSA approach via the g_mmpbsa utility [55]. Finally, for the last 50 ns of the 4CJN-ITC and 4CJN-CCD, binding free energy was estimated every 1000 frames, providing information on ligand stability and binding affinity.

This method enables the direct computation of the binding energy of the end states, thereby eliminating the need for intermediate state simulations. The molecular mechanical energies were integrated with continuum solvent models, and explicit water molecules were removed from the trajectories to facilitate the calculation. Further, we also performed principal component analysis (PCA) and free energy landscapes (FELs) of the two complexes. FEL can be very useful in studying the folding mechanism of proteins and their structural stability, while PCA is a widely used statistical technique for the analysis of large-scale atomic motions and identification of the major conformational changes in biomolecular systems. PCA mainly focused on the first several eigenvectors (EVs) as these have been shown to correspond to the significant global motions of the protein.

## 3. Results

### 3.1. Chemistry

**Scheme 1:**
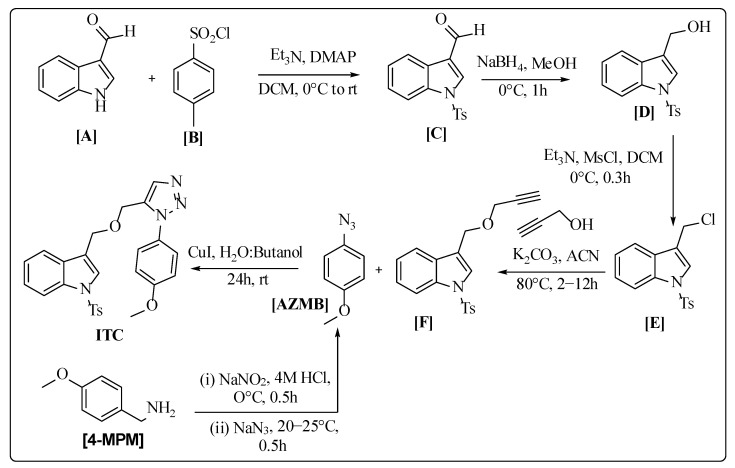
Synthesis of 3-(((2-(4-methoxyphenyl)-2H-1,2,3-triazol-4-yl) methoxy)methyl)-1-tosyl-1H-indole (ITC).

The ITC was synthesized using Scheme 1. The scheme was developed based on references from Chen et al. (2016), Banday et al. (2010), Yele et al. (2021), Liu et al. (2003), and Siddiki et al. (2013) [56,57,58,59,60]. The compound ITC was prepared by the click reaction using green chemistry. The ITC was purified through CC (ethyl acetate/hexane mixture (6:4)). The Fourier-Transform Infrared (FT-IR) spectrum of ITC exhibited characteristic SO_2_ stretching vibrations at 1165 cm^−1^ and 1136 cm^−1^, while C-H symmetric and asymmetric vibration peaks of indole appeared in the 3025 and 3045 cm^−1^ regions. The appearance of characteristic C-H stretching of the triazole ring at 3106 cm^−1^ and the band of O-C at 1239 cm^−1^ supported the development of the compound ITC.

The structural elucidation of the ITC was conducted using proton nuclear magnetic resonance (^1^H-NMR) (Figure S1), carbon-13 nuclear magnetic resonance (^13^C-NMR) (Figure S2), and high-resolution mass spectrometry (HRMS) (Supplementary Data, Figure S3).

In the ^1^H-NMR spectrum of ITC, the characteristic -CH- proton signal of the triazole moiety was observed at δ 8.71 ppm. The two -CH_2_ protons were observed as distinct singlets at δ 4.72 and 4.674 ppm, while the -OCH_3_ proton was detected as a singlet at δ 3.84 ppm. Additionally, the singlet peak at δ 2.30 ppm was attributed to the -CH_3_ group of ITC. The spectrum revealed aromatic proton resonances in the range δ 7.93–7.13 ppm, allowing for the assignment of coupling constants (J values) and chemical shifts based on the position and substitution pattern of these protons. The ortho, meta, and para protons displayed distinctive splitting patterns, including singlets, doublets, and triplets, which reflected their unique electronic environments and coupling interactions.

In the ^13^C-NMR spectrum, the C-O-C signal of ITC appeared as a singlet within the range of δ 169.42–162.41 ppm, while aromatic carbon signals were observed in their expected region. The characteristic -C-N peak was detected at 159.72 ppm. Additionally, the signals corresponding to two CH_2_ groups, an OCH_3_ group, and a CH_3_ group were observed at δ 63.42, 63.10, 56.10, and 22.02 ppm, respectively. The HRMS data further confirmed the molecular formula of **ITC**, validating its structural composition.

### 3.2. In Vitro Analysis

#### 3.2.1. MIC_50_ Analysis

Indoles and their derivatives have been well-documented for synthetic versatility and diverse biological properties. However, specific antibacterial targets of indole conjugated with other heterocyclic moieties remain to be fully elucidated through detailed biological studies. This study assessed the antibacterial efficacy of ITC against clinically significant bacterial strains, with ciprofloxacin serving as the reference antibiotic (Table 1). MIC_50_ values were determined in accordance with CLSI guidelines, utilizing the broth microdilution method. The results indicated that ITC exhibited varying degrees of antibacterial activity, with MIC_50_ values ranging from 0.115 ± 0.021 to 0.998 ± 0.053 μg/mL against bacterial strains. Specifically, ITC demonstrated variable activity against GNB, such as EC (0.639 ± 0.018 μg/mL), PA (0.766 ± 0.016 μg/mL), and ST (0.564 ± 0.045 μg/mL). In contrast, it exhibited significant activity against GPB such as BS (0.347 ± 0.036 μg/mL), SE (0.443 ± 0.027 μg/mL), and SA (0.368 ± 0.036 μg/mL). Notably, ITC displayed potent activity against MRSA strains (NCIM 5021: 0.156 ± 0.003 μg/mL) and (ATCC 43300: 0.115 ± 0.021 μg/mL), demonstrating enhanced efficacy against resistant strains compared to wild-type bacterial strains. Substitution on the C-3 position with sulphur and triazole moiety, which acts as a linker between heteroaryl and aryl rings, might have resulted in improved inhibitory activity.

#### 3.2.2. MBC Analysis

The MBC of the ITC was determined following standard CLSI guidelines using ciprofloxacin as a standard. A compound is bactericidal if the MBC/MIC_50_ ratio is ≤2, whereas a ratio of ≥4 indicates it is bacteriostatic [61]. The MBC values of ITC were determined to be 1–4 times the MIC_50_ against the tested bacterial strains, as presented in Table 1. Notably, ITC demonstrated potent bactericidal activity against GP bacteria at concentrations equivalent to the MIC_50_, whereas its efficacy against GN bacteria was relatively reduced. Substitution on the C-3 position with sulphur and triazole moiety, which acts as a linker between heteroaryl and aryl rings, and also a tosyl moiety on the indole, might have resulted in enhanced inhibitory activity. These findings suggest that ITC exhibits potent bactericidal activity, highlighting its potential as a promising antibacterial agent.

#### 3.2.3. Time–Kill Study

**Figure 2:**
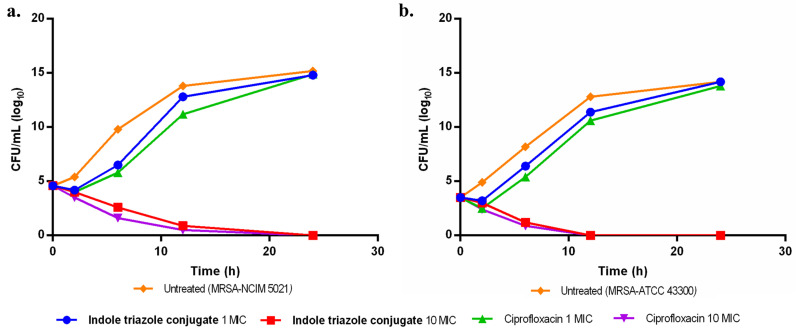
Illustrates the time–kill curve of ITC against (**a**) MRSA (NCIM 5021), (**b**) MRSA (ATCC 43300).

The bactericidal activity of ITC was evaluated based on MIC_50_ and MBC assay results, which were further validated using a time–kill study. The results demonstrated that ITC exhibited significant bactericidal activity against both resistant strains, effectively reducing bacterial counts compared to the untreated control (Figure 2a,b). However, from the study, it is also clear that ITC is bactericidal against MRSA (ATCC43300) as compared to MRSA (NCIM5021). Thus, further studies were carried out against MRSA (ATCC43300).

#### 3.2.4. Cytotoxicity Study

The cell toxicity of ITC was assessed using the MTT assay on Vero cells to assess its effect on mammalian cells. In this study, doxorubicin was used as a reference standard, and all experiments were accomplished in triplicate to ensure accuracy and reproducibility. Cytotoxicity analysis revealed that ITC exhibited no significant toxicity against Vero cells, with a CC_50_ value of >5 µg/mL (Table 2). Furthermore, ITC demonstrated a promising selectivity index (SI), with values of >43.67 µg/mL against MRSA (ATCC 43300) and >32.05 µg/mL against MRSA (NCIM 5021), indicating its potential therapeutic efficacy with minimal cytotoxic effects. These findings indicate that ITC possesses a favorable therapeutic window, demonstrating potent antibacterial activity while maintaining low cytotoxicity.

#### 3.2.5. Synergy Testing

In this study, a standard checkerboard assay was conducted to assess pharmacological interactions between ITC and various FDA-approved antibiotics (ciprofloxacin, gentamicin, linezolid, daptomycin, and rifampicin). This model allowed the interactions to be classified as synergistic, additive, or antagonistic and provides insight into the therapeutic potential of antibiotic combination regimens against antibiotic-resistant bacterial strains. Overall, synergistic interaction was observed with ITC and antibiotics, as shown in Table 3, which emphasizes the potential of ITC in multidrug therapy against resistant strains.

#### 3.2.6. Post-Antibiotic Effect (PAE)

The PAE of ITC was assessed using Ciprofloxacin and Gentamicin as controls against MRSA (ATCC43300). As shown in Table 4, ITC displayed a PAE of 3 h at 1× MIC_50_ and 4 h at 10× MIC_50_, demonstrating a comparable duration of bacterial suppression to that of ciprofloxacin and gentamicin. These findings suggest that ITC maintains prolonged antibacterial activity, which could contribute to its potential effectiveness in antimicrobial therapy.

#### 3.2.7. Biofilm Activity

Bacteria have been shown to form biofilms as a protective mechanism under various stress conditions, which often complicates therapeutic interventions and contributes to increased drug resistance. Notably, most approved antibiotics exhibit limited efficacy against biofilm-associated pathogens due to their altered physiological state. Therefore, assessing the antibiofilm activity of newly developed molecules is crucial for identifying effective therapeutic candidates.

**Figure 3:**
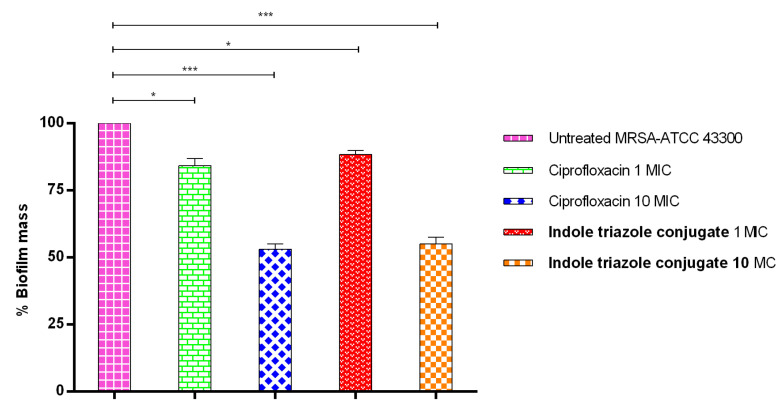
Illustrates the activity of ITC against MRSA (ATCC43300) pre-formed biofilm. *p* < 0.05 (*), *p* < 0.001 (***).

In this study, the ITC demonstrated significant antibiofilm activity at 10× MIC_50_, reducing biofilm formation by 45% compared to untreated MRSA. A similar level of biofilm reduction (47%) was observed with ciprofloxacin at the same concentration (10× MIC_50_). The present findings demonstrate that ITC possesses equivalent efficacy to ciprofloxacin in preventing biofilm formation, highlighting its therapeutic potential as an effective antibiofilm agent (Figure 3).

### 3.3. Computational Analysis

#### 3.3.1. Molecular Docking

Docking was conducted using the X-ray crystal structure of PBP2a (PDB ID: 4CJN), a representative bacterial macromolecular enzyme involved in MRSA resistance. The compound ITC demonstrated a strong binding affinity to the allosteric site of PBP2a, signifying its potential as an effective inhibitor of this target.

**Figure 4:**
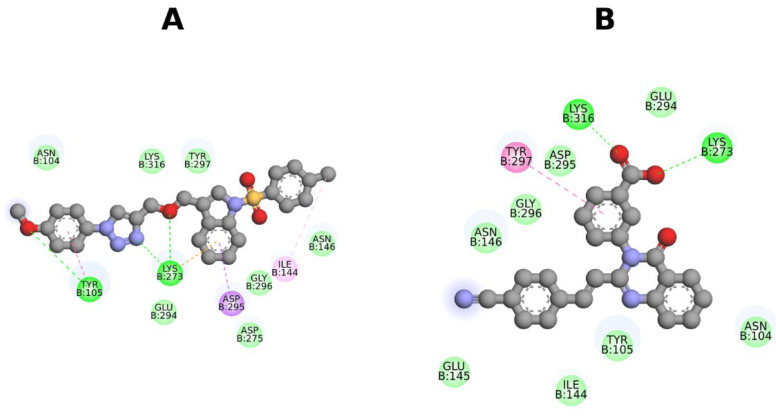
The 2D images of the complex interactions of (**A**) 4CJN-ITC, (**B**) 4CJN-CCD.

In the current molecular docking study, the 4CJN-ITC complex exhibited a docking score of −6.7 kcal/mol, demonstrating the strongest binding affinity at the allosteric site. The findings were compared to those of CCD (−6.3 kcal/mol), suggesting that ITC might engage in stable interactions with the receptor (Table 5). The complex interacts with a broad array of key residues, including Asn104, Tyr105, Ile144, Asn146, Lys273, Asp275, Glu294, Asp295, Gly296, Tyr297, and Lys316, indicating a stable and more effective binding mode (Figure 4a). All these interactions suggest that the ligand adopts a favorable conformation, facilitating significant binding to the 4CJN. With a docking score of −6.3 kcal/mol obtained for the CCD using the same docking protocol, this value was used as a reference to evaluate the relative binding affinity of the ITC. While it interacts with important residues like Asn104, Tyr105, Ile144, Glu145, Asn146, Lys273, Glu294, Asp295, Gly296, Tyr297, and Lys316, the interaction is comparatively stronger, possibly due to fewer interactions or a less optimal binding orientation (Figure 4b).

#### 3.3.2. MD Analysis

MD simulations have become a powerful means of elucidating the structural dynamics of the receptor and its interactions with the ligand. The study was performed to characterize the conformational changes generated by the target protein–ligand association. The structural and dynamic parameters, like RMSD, RMSF, Rg, SASA, and intermolecular hydrogen bonding, have been evaluated for 4CJN–ITC and 4CJN–CCD complexes. These analyses provide data on the stability, flexibility, and compactness of the complexes and aid in the characterization of their intermolecular interactions.

(i) RMSD

**Figure 5:**
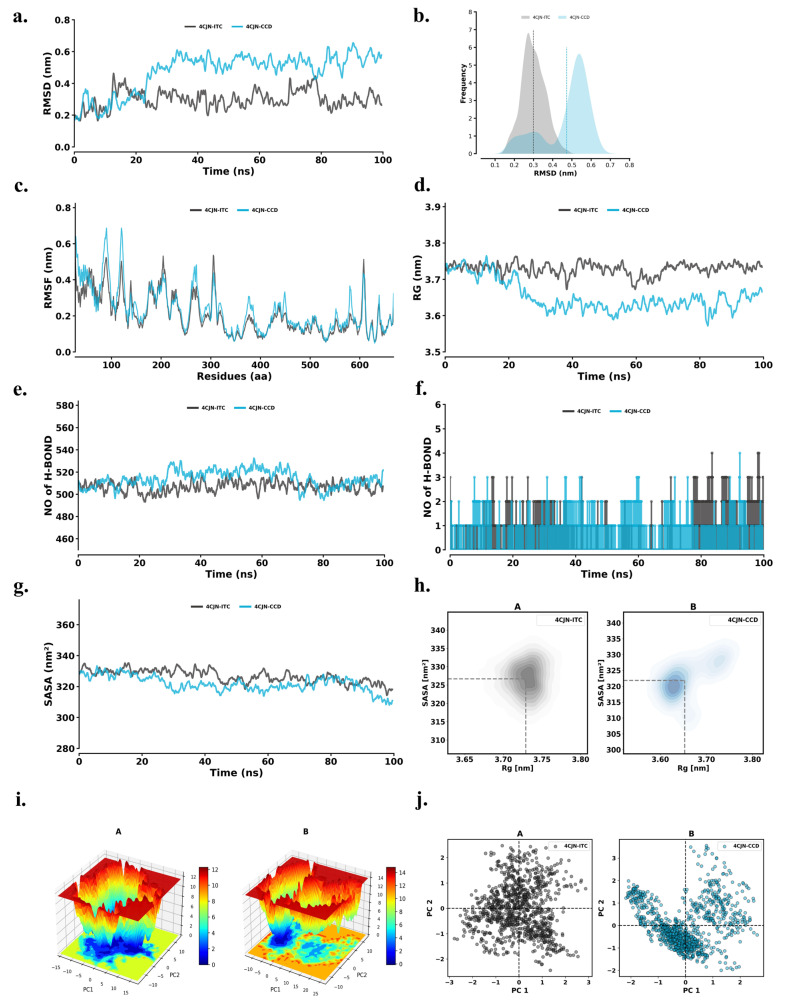
(**a**) RMSD conformational dynamics; (**b**) PD plot; (**c**) RMSF analysis; (**d**) Rg analysis; (**e**) intramolecular hydrogen bonds; (**f**) intermolecular hydrogen bonds; (**g**) SASA analysis; (**h**) kernel density estimation plot of conformational distribution based on Rg and SASA values; (**i**) free energy landscape plots; (**j**) principal component analysis of 4CJN-ITC (A: black dots) and 4CJN-CCD (B: blue dots).

For this analysis of the protein–ligand complexes, a time-dependent analysis of the RMSD (root mean square deviation) values was performed (Figure 5a). The results suggest that both systems reached equilibrium within 25 ns and were stable during the 100 ns simulation timeline. The calculated root mean square deviation (RMSD) indicated low deviations among the 4CJN-ITC and 4CJN-CCD complexes, suggesting that these complexes can maintain their structural consistency. This indicates that the structure of the obtained complexes was preserved with RMSD values (average RMSD = 0.30 ± 0.06 nm for 4CJN-ITC and 0.47 ± 0.12 nm for 4CJN-CCD). The root mean square deviation (RMSD) of 4CJN-ITC was also lower than 4CJN-CCD, which substantiates lesser conformational fluctuations in the case of 4CJN-ITC, denoting a more stable binding interaction. This implies that the 4CJN-ITC complex is more stable than 4CJN-CCD.

RMSD for the CCD and ITC complexes represented in Figure 5b provides information on the structural stability and conformational flexibility. The graph shows that 4JCN-CCD has a wider RMSD distribution with a peak at around 0.48 nm, implying that it has more conformational variability. However, the 4JCN-ITC has an RMSD value between 0.1 and 0.5 nm, with a peak density near 0.3 nm, as shown in the probability plots. The results revealed that the RMSD distribution of 4JCN-ITC was narrower than that of 4JCN-CCD, indicating that the 4JCN-ITC complex is more rigid and stable in comparison to CCD, which has a wider distribution, suggesting more flexible movement.

(ii) **RMSF**

RMSF is an important metric for assessing the flexibility of amino acid residues and finding dynamic regions in the target. Moreover, RMSF values give further insight into how the binding of a ligand affects the stability of the protein. Compact structural motifs like β-sheets and α-helices generally display lower RMSF values, while flexible loop regions are characterized by greater fluctuations.

We measured the RMSF for residues in both 4CJN-ITC and 4CJN-CCD complexes and plotted the data in Figure 5c for residue-specific conformational flexibility/binding dynamics. The average RMSF for 4CJN-ITC was 0.21 ± 0.11 nm, while 4CJN-CCD was 0.23 ± 0.12 nm. The overall distribution of the RMSF, indicating the stability and flexibility of the protein structure, was preserved in the two complexes, implying that ligand binding did not influence significant structural conformational changes. The similar distributions of RMSFs in the two complexes indicate that the intrinsic flexibility of the protein is retained after ligand binding, which may be important for its biological activity.

(iii) Rg

Rg values were calculated with respect to the time for the 4CJN-ITC and 4CJN-CCD complexes to estimate the dynamic stability and compactness of these complexes (Figure 5d). The Rg averaged 3.73 ± 0.02 nm for 4CJN-ITC and 3.65 ± 0.04 nm for 4CJN-CCD. Rg profiles were similar for both complexes, suggesting similar compactness of the structure. These constant Rg values suggest that ITC/CCD binding to 4CJN does not result in any significant conformational change or destabilization of the protein. Compactness preservation is important for maintaining the functional integrity of the protein, promoting the stability of the complexes even more.

(iv) Intra and Inter Hydrogen Bond

Intramolecular and intermolecular hydrogen bond formation is a major factor determining the stability of a protein with respect to structure and ligand binding. The time-dependent behavior of intra- (Figure 5e) and intermolecular (Figure 5f) hydrogen bonds was analyzed for 4CJN-ITC and 4CJN-CCD complexes. Intra-hydrogen bonds were 506.94 ± 10.46 in the 4CJN-ITC complex and 513.96 ± 11.98 in the 4CJN-CCD complex. These results suggest that ITC and CCD binding to 4CJN did not substantially alter the internal hydrogen bonding network of the protein, which is known to play an important role in maintaining the structural stability and integrity of proteins. In summary, the preservation of these key contacts indicates the general conformational background of 4CJN is maintained, adding further weight to the stabilities of the complexes. Likewise, the docked complexes remained stable across the simulation, with 4CJN-ITC only forming one to six intermolecular hydrogen bonds and 4CJN-CCD maintaining one to four intermolecular hydrogen bonds. Therefore, the large number of hydrogen bonds formed in the 4CJN-ITC complex confirms the intermolecular stabilizing effect that can lead to strong binding and better structural retention.

(v) SASA

The surface area of the solvent accessible (SASA) was used for calculation to analyze the effect of ligand binding on the target protein’s solvent exposure. SASA, one of the most important parameters, gives vital insight into the accessibility of the protein in a solvent environment to illustrate the binding-induced alterations in protein structure and function (Figure 5g). These results showcased a similar tendency for SASA value between 4CJN-ITC and 4CJN-CCD, suggesting similar solvation behavior. The SASA (solvent accessible surface area) values were 326.71 ± 4.74 for 4CJN-ITC and 321.87 ± 5.48 for 4CJN-CCD. SASA remained almost constant during the simulation, indicating that the complexes were well solvated. Such stability is pivotal for deciphering their thermodynamic and kinetic behavior and provides an additional layer of structural coherence to the complexes. Further, to assess the compactness and conformational behavior of the complexes, Keller density estimation plots were created to visualize the relationship between Rg and SASA throughout the simulation study (Figure 5h). The 4CJN-ITC complex established a relatively tight clustered density (Rg ~ 3.70 nm and SASA ~ 325–330 nm^2^), suggesting stable and compact conformations with minimal fluctuation, whereas 4CJN-CCD exhibited a broader distribution in both the Rg and SASA, indicating greater flexibility and a high degree of conformational sampling. These findings are in line with the RMSD and RMSF results, where ITC binding contributed to a more stable and compact 4CJN conformation, whereas the CCD complex displayed fair dynamic behavior.

(vi) Free Energy Landscapes (FELs)

FEL can be very useful in studying the folding mechanism of proteins and their structural stability. Figure 5i describes FEL plots shown for the PC1 and PC2. In addition to this, FEL plots allow us to understand not just the fold itself, but also the configuration of the most stable conformations of the protein (the basin of energy wells, in which lower energy wells are considered more stable states). The darker blue areas in the plots represent low-energy conformations, or the most stable conformers. The energy values during the simulation for the 4CJN-ITC complex were uniform between 0 to 14 kJ/mol and 0 to 16 kJ/mol for the 4CJN-CCD complex. Results from the FEL analysis revealed that the 4CJN-CCD complex has only one global minimum, along with a large local basin, indicating a stable conformational state. The binding of 4CJN-CCD does not induce large-scale conformational changes with respect to their overall structures, indicating that the structural stability of the target protein remains unchanged. The analysis with FEL reveals the energetic stability and conformational diversity of the two complexes, showcasing the relevance of energy landscapes to understand protein–ligand interactions.

(vii) Principal Component Analysis (PCA)

To evaluate the conformational dynamics of 4CJN-ITC and 4CJN-CCD during the simulation, PCA analysis was performed (Figure 5j). These results reveal the structural ensembles and functional motions of the complexes, which further validate their compositions. PCA across time indicated a decreased flexibility of both the complexes in the principal components, consistent with enhanced arrangement stability. PCA plots demonstrate that 4CJN-ITC and 4CJN-CCD complexes explored nearly equivalent conformational space, with considerable overlap in terms of their movement patterns. Fewer large-scale movements or decay in 4CJN-CCD, suggesting a complex perturbed minimal perturbation to the native conformational space. The reduced variance suggests that the complex adopts a more rigid and stable conformation, potentially contributing to enhanced structural integrity.

(viii) Protein–Ligand Interactions

**Figure 6:**
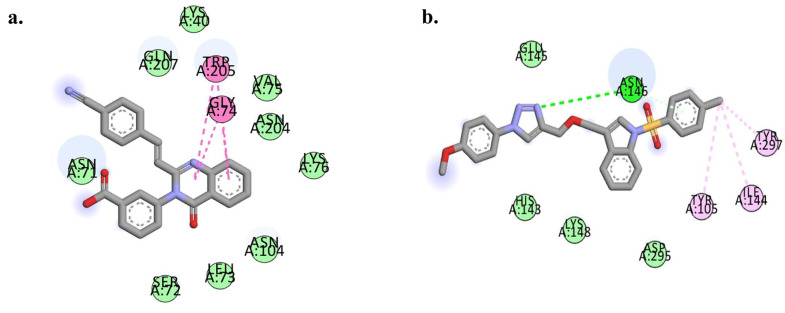
Representative 2D interaction diagrams showing molecular dynamics-derived binding interactions of the (**a**) 4CJN-CCD and (**b**) 4CJN-ITC complexes.

Protein–ligand interactions for the ITC and CCD were generated to obtain insights into the residue-level binding stability during the MD study (Figure 6a,b). The ITC possessed stable hydrogen bonding interactions with Asn146. π–π stacking interactions observed for residues Tyr105, Ile144, and Tyr297. These ligand contacts with the receptor represent a robust and spatially favorable binding mode, which was further supported by dynamic stability observed through RMSD and PCA analysis. In contrast, CCD exhibited key interactions with Gly74 and Trp205, suggesting a stable π–electron-mediated anchoring of the compound within the binding pocket. The broader interaction network and dual hydrophilic-hydrophobic contacts observed for ITC reflect better binding complementarity and contribute to its enhanced dynamic stability compared to CCD.

#### 3.3.3. MM-PBSA

Using the MM-PBSA method, the relative binding affinity and free energy of the 4CJN-ITC and 4CJN-CCD complexes were calculated. Table 6 represents the comparison of the binding strengths of the ITC and CCD with the target protein. A residue-level energy decomposition analysis was conducted over a stable simulation trajectory to identify critical residues responsible for complex stability. These analyses indicate the detailed dynamics of the molecular interactions at the core of ligand binding and complex stability, including the specific residues responsible for these processes.

Although our computational studies indicate that ITC binds to the allosteric site of PBP2a (as observed with CCD in PDB ID: 4CJN). However, the hypothesis requires experimental validation through direct biochemical or genetic evidence to confirm PBP2a as the primary molecular target. Therefore, further mechanistic studies, like competitive binding assays or resistance profiling, are necessary to validate this interaction and confirm the mode of action.

## 4. Conclusions

In conclusion, a new compound of 3-(((2-(4-methoxyphenyl)-2H-1,2,3-triazol-4-yl) methoxy)methyl)-1-tosyl-1H-indole (ITC) was synthesized, and its structure was characterized and tested for antibacterial activity. ITC showed significant MIC_50_ (0.115 ± 0.021 µg/mL), MBC (0.11 ± 0.021 µg/mL), and time–kill kinetics against the MRSA strain. In addition, ITC possessed potent PAE (2 h > gentamicin, ciprofloxacin), together with a concentration-dependent bactericidal effect. Moreover, ITC exhibited synergistic action with FDA-approved drugs, supporting its potential for multi-drug therapy. ITC also showed remarkable antibiofilm activity, evidenced through the substantial decrease in their biofilm biomass, and also stands out as a potential chemical backbone having potential application in anti-bacterial treatment strategies. Based on observed activity, PBP2a, a key resistance determinant in MRSA, was selected for computational evaluation. The compound ITC exhibited favorable binding affinity towards the target protein, as evident by its docking score (−6.7 kcal/mol) and dynamic simulation studies. This selective inhibition of PBP2a supports its macromolecular mechanism of action and underscores the compound’s relevance in tackling multidrug-resistant pathogens. The target of ITC, a representative compound, is the antibiotic-resistant bacterial strains, making it an attractive target to develop novel antibacterial therapeutics. However, we acknowledge that no direct mechanistic studies were conducted to confirm the mode of action, and the target specificity remains to be validated. Due to the structural flexibility of small molecules like ITC, off-target interactions or promiscuity cannot be ruled out at this stage. Future studies include enzyme inhibition, target validation, and omics-based approaches are needed to confirm the exact mechanism and specificity. Overall, ITC emerges as a potential scaffold for further development, with future work focusing on structure-activity optimization and target validation.

## 5. Patent

The novel compound Indole Triazole Conjugate (ITC) was patented under Indian Product Patent with Application No: 202141003113.

## Figures and Tables

**Table 1:** MIC_50_ and MBC of ITC against selected panel of bacteria.

Organisms	MIC_50_ (μg/mL)		MBC (μg/mL)		MIC_50_/MBC Ratio	
	ITC	CIP	ITC	CIP	ITC	CIP
*E. coli*	0.639 ± 0.018	0.468 ± 0.014	1.253 ± 0.007	0.441 ± 0.041	2	1
*P. aeruginosa*	0.766 ± 0.016	0.595 ± 0.052	1.565 ± 0.186	0.551 ± 0.033	2	1
*K. pneumonia*	0.564 ± 0.045	0.616 ± 0.007	1.247 ± 0.273	0.565 ± 0.041	2	1
*S. typhi*	0.872 ± 0.019	0.732 ± 0.020	1.686 ± 0.010	0.706 ± 0.011	2	1
*B. subtilis*	0.347 ± 0.036	0.460 ± 0.035	0.325 ± 0.045	0.434 ± 0.047	1	1
*S. epidermis*	0.443 ± 0.027	0.354 ± 0.038	0.441 ± 0.055	0.561 ± 0.310	1	1
*S. aureus*	0.368 ± 0.036	0.249 ± 0.036	0.347 ± 0.019	0.241 ± 0.009	1	1
MRSA (NCIM 5021)	0.156 ± 0.003	0.256 ± 0.041	0.167 ± 0.028	0.237 ± 0.168	1	1
MRSA (ATCC43300)	0.115 ± 0.021	0.331 ± 0.035	0.111 ± 0.021	0.371 ± 0.017	1	1

MIC_50_: Minimum inhibitory concentration; MBC: Minimum bactericidal concentration; ITC: Indole triazole conjugate; CIP: Ciprofloxacin.

**Table 2:** Cytotoxicity profile and selectivity index (SI) of ITC.

Strain	MIC_50_ (μg/mL)	CC_50_ (μg/mL)	Selectivity Index (CC_50_/MIC_50_)
MRSA (ATCC 43300)	0.115 ± 0.021	>5	>43.47
MRSA (NCIM 5021)	0.156 ± 0.003	>5	>32.05

MIC_50_: Minimum inhibitory concentration; CC_50_: Cell cytotoxicity; MRSA: Methicillin-resistant *Staphylococcus aureus*.

**Table 3:** Synergy testing of ITC.

Drug	MRSA (ATCC 43300)	MIC_50_ of ITC in the Presence of Drug (A)	MIC_50_ of Drug in the Presence of ITC (B)	FIC A	FIC B	∑FIC A + FIC B	Inference
ITC	0.368						
Ciprofloxacin	0.249	0.0098	0.00195	0.0266	0.0078	0.0344	synergistic
Gentamicin	0.278	0.0256	0.00245	0.0695	0.0088	0.0783	synergistic
Linezolid	1.896	0.0078	0.00912	0.0211	0.0048	0.0259	synergistic
Daptomycin	0.988	0.0985	0.00078	0.2676	0.0007	0.2683	synergistic
Rifampicin	0.237	0.0781	0.00913	0.2122	0.0385	0.2507	synergistic

MIC_50_: Minimum inhibitory concentration; ITC: Indole triazole conjugate; MRSA: Methicillin-resistant *Staphylococcus aureus*; FIC: Fractional inhibitory concentration.

**Table 4:** Post-antibiotic effect of ITC.

Treatments	Time for 1 log10 (h)	PAE = T − C
MRSA ATCC43300	2	0
ITC 1× MIC_50_	3	−1
ITC 10× MIC_50_	4	−2
CIP 1× MIC_50_	3	−1
CIP 10× MIC_5_	4	−2

ITC: Indole triazole conjugate; CIP: Ciprofloxacin.

**Table 5:** Docking scores and interactions with the protein 4CJN.

Complex	Docking Score (kcal/mol)	Interactions
4CJN-ITC	−6.7	Asn104, Tyr105, Ile144, Asn146, Lys273, Asp275, Glu294, Asp295, Gly296, Tyr297, Lys316
4CJN-CCD (Cocrystal)	−6.3	Asn104, Tyr105, Ile144, Glu145, Asn146, Lys273, Glu294, Asp295, Gly296, Tyr297, Lys316

ITC: Indole triazole conjugate; CIP: Ciprofloxacin.

**Table 6:** MM-PBSA analysis of ITC.

Complexes	Van der Waals Energy	Electrostatic Energy	Polar Solvation Energy	Binding Energy
4CJN-ITC	−107.042 ± 26.010 kJ/mol	−9.905 ± 19.250 kJ/mol	73.494 ± 26.797 kJ/mol	−55.507 ± 24.610 kJ/mol
4CJN-CCD	−132.209 ± 28.286 kJ/mol	−1.847 ± 11.742 kJ/mol	99.163 ± 24.527 kJ/mol	−50.128 ± 22.690 kJ/mol

## Data Availability

Data provided in supplementary.

## Supplementary Materials

The following supporting information can be downloaded at: https://www.mdpi.com/article/10.3390/pharmaceutics17081013/s1, Figure S1: Proton NMR of ITC; Figure S2: Carbon NMR of ITC; Figure S3: HRMS spectra of ITC.

## Abbreviations

The following abbreviations are used in this manuscript:

ABR	Antibacterial resistance
MRSA	Methicillin-resistant *Staphylococcus aureus*
PBP2a	Penicillin-binding protein 2a
ITC	Indole triazole conjugate
PDB	Protein Data Bank
FEL	Free energy landscape
PCA	Principal component analysis
MD	Molecular dynamics simulation
