# Supplementary material for: Synthesis and Antibacterial Evaluation of an Indole Triazole Conjugate with In Silico Evidence of Allosteric Binding to Penicillin-Binding Protein 2a
Source: Pharmaceutics. 2025 Aug 3;17(8):1013. doi: 10.3390/pharmaceutics17081013 (PMC12389187; doi:10.3390/pharmaceutics17081013)
Supplement: Supplementary file 1 [file pharmaceutics-17-01013-s001.zip › pharmaceutics-3741109-supplementary.pdf]

Supplementary Data

Synthesis and Antibacterial Evaluation of an Indole Triazole Conjugate with In Silico Evidence of Allosteric Binding to Penicillin-Binding Protein 2a

Vidyasrilekha Sanapalli [1,2,*], *Bharat Kumar Reddy Sanapalli* [3,4], *Afzal Azam MD* [1,5]

[1] Department of Pharmaceutical Chemistry, JSS College of Pharmacy, JSS Academy of Higher Education & Research, Ooty, The Nilgiris, Tamil Nadu 643001, India

[2] Department of Pharmaceutical Chemistry, School of Pharmacy & Technology Management, SVKM's Narsee Monjee Institute of Management Studies (NMIMS) Deemed-to-be-University, Jadcherla - 509301, Hyderabad, India.

[3] Department of Pharmaceutics, JSS College of Pharmacy, JSS Academy of Higher Education & Research, Ooty, The Nilgiris, Tamil Nadu 643001, India

[4] Department of Pharmacology, School of Pharmacy & Technology Management, SVKM's Narsee Monjee Institute of Management Studies (NMIMS) Deemed-to-be-University, Jadcherla - 509301, Hyderabad, India.

[5] Department of Pharmaceutical Chemistry, JSS College of Pharmacy, JSS Academy of Technical Education, Noida, Uttar Pradesh 201301, India

Corresponding author*

Dr. Vidyasrilekha Sanapalli, M. Tech (Pharma)., Ph.D.

E-mail: vidyasrilekha16@gmail.com

Assistant Professor, Department of Pharmaceutical Chemistry,

School of Pharmacy & Technology Management,

SVKM's Narsee Monjee Institute of Management Studies (NMIMS) Deemed-to-be-University, Jadcherla - 509301, Hyderabad, India

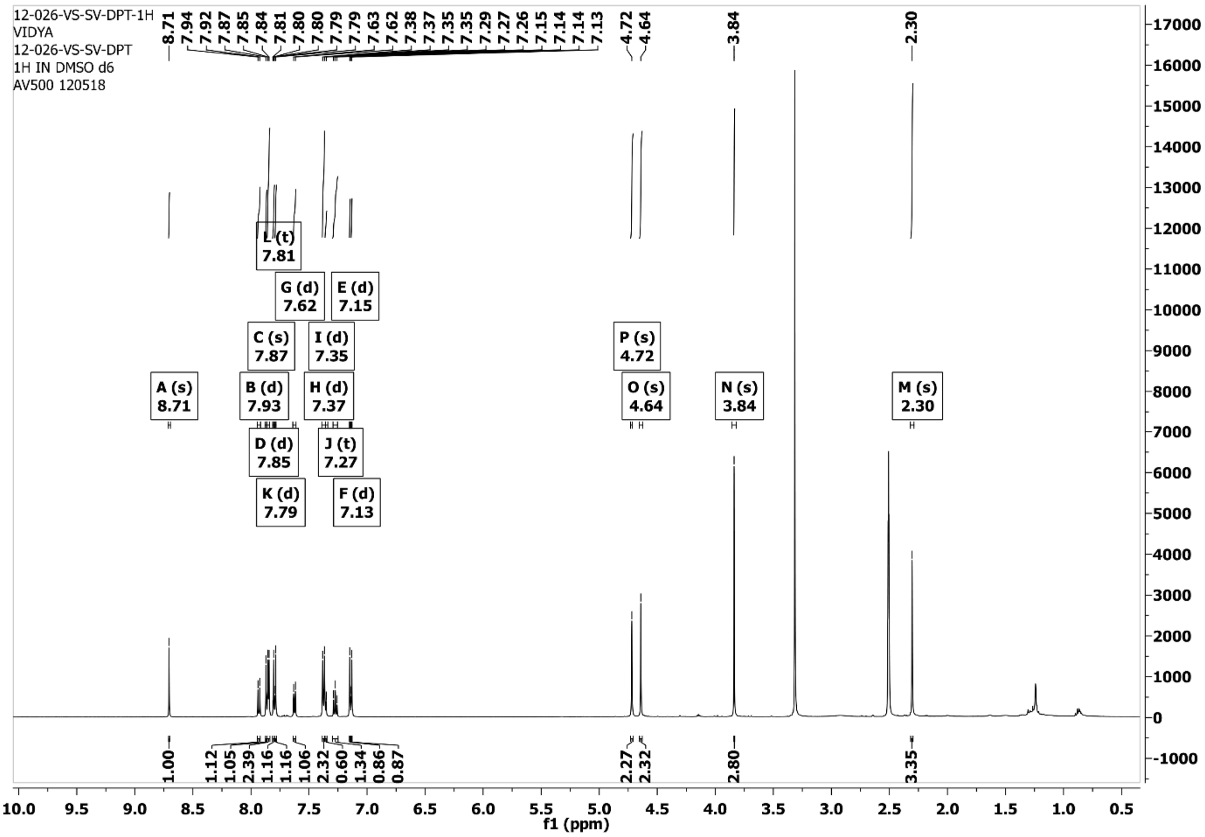

Figure S1: Proton NMR of ITC

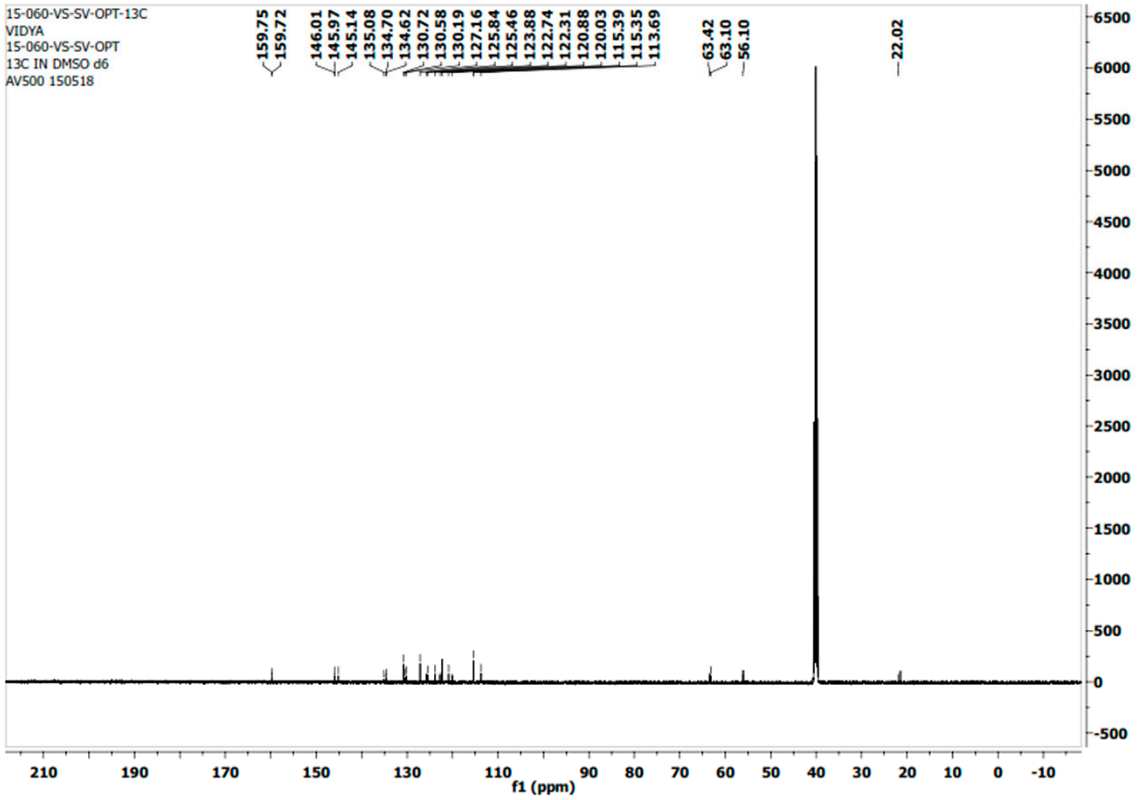

Figure S2: Carbon NMR of ITC

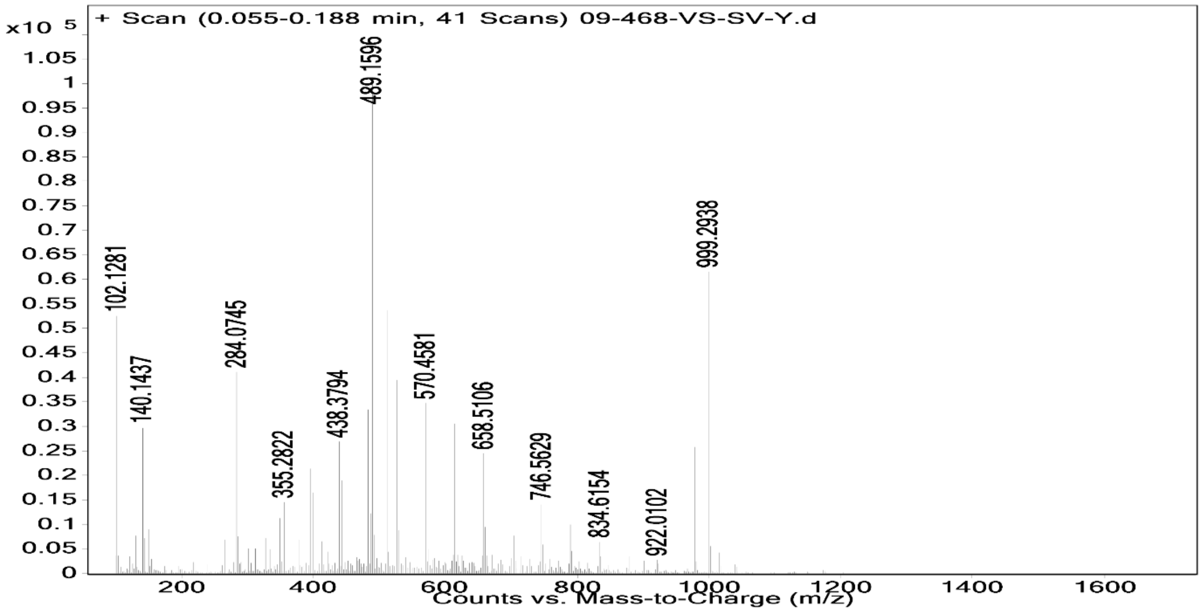

Figure S3: HRMS spectra of ITC
